# The hidden costs of emotional labor on withdrawal behavior: the mediating role of emotional exhaustion, and the moderating effect of mindfulness

**DOI:** 10.1186/s40359-023-01392-z

**Published:** 2023-10-18

**Authors:** Peng Peng, Xintian Li

**Affiliations:** 1 School of Business, Qingdao University of Technology, Qingdao, China; 2 School of Business, Shandong Jianzhu University, Jinan, China

**Keywords:** Emotional labor, Emotional exhaustion, Withdrawal behavior, Mindfulness

## Abstract

**Background:**

Employees’ withdrawal behavior concerns organization leaders and policymakers in many countries. However, the specific mechanism which emotional labor affects withdrawal behavior has yet to be thoroughly discussed. There needs to be systematic research on how different emotional labor strategies affect work withdrawal, whether directly or through individual perception, and how to respond.

**Methods:**

A total of 286 hotel and catering service employees participated in our study. A series of hierarchical moderated regression analyses were performed to test the hypothesis.

**Results:**

The results indicated that surface acting positively affected withdrawal behavior, while deep acting had a negative effect. Emotional exhaustion mediated in this relationship of surface acting with withdrawal behavior and deep acting with withdrawal behavior. Mindfulness showed moderation effects between emotional exhaustion and withdrawal behavior.

**Conclusions:**

Emotional labor and emotional exhaustion are significant in predicting employees’ intentions to withdraw, given that emotional exhaustion partially mediates the effects of emotional labor on withdrawal behavior. Significantly, the relationship between emotional exhaustion and withdrawal behavior is weakened by mindfulness.

## Introduction

The term quiet quitting was popularized in 2022, which means that when physically, people still turn up to work but mentally check out and do the bare minimum to get [1]. This approach to work encourages employees to perform their duties without fully agreeing with a “work is life” culture, thus leading their careers and standing out to their managers. It is not unique; the word “Lying Flat” is trending in China right now, connoting that no matter how hard you work, you won’t achieve your once-in-a-lifetime goals, so you better become a “couch potato.“ Quiet quitting is a new name for an old method of industrial action [1]. Although the term quiet quitting comes from a younger generation and has new packaging, it has been used for decades to study this phenomenon under different names: disengagement, neglect, and withdrawal, so drawing on the concept of work withdrawal behavior research has implications for understanding and managing quiet quitting.

Emotional labor is a unique mode in which employees show their emotional disclosure in line with organizational norms and work needs and then complete their work requirements [2]. Emotional labor is an essential feature of the service industry. Service organizations, without exception, require employees to show appropriate emotions in their work. Therefore, employees will adjust, control, or change their inner emotional state to adapt to their emotional expression needs. Emotional labor is the emotional management strategy adopted by people at work. Whether employees can adopt effective emotional management strategies at work and have enough ability to carry out emotional labor significantly correlate with their psychological state. Studies have pointed out that employees using emotional labor strategies will cause them to experience more job burnout, and employees who undertake excessive emotional labor may show a tendency to leave. Emotional labor significantly impacts employees’ attitudes, behavior, corporate image, customer experience, etc. First, emotional labor requires employees to cover up and suppress their emotions for a long time and needs to consume specific emotional resources. If this re-source consumption cannot be supplemented in time, it will produce physical and mental fatigue and work fatigue, leading to withdrawal behavior at work. Emotional labor has a negative impact on employees mainly from the surface effect, which brings employees to emotional exhaustion [3], negative emotions [4], work burnout [2], work turnover intention [5], etc. Secondly, emotional labor will also have an impact on customers. Employees do emotional labor to meet customer expectations and organizational requirements. However, if customers realize that employees’ emotions are disguised and false, they will negatively evaluate their services and cause reduced satisfaction [6].

Work withdrawal behavior caused by emotional labor is a problem that organizations must pay great attention to. However, the specific mechanism which emotional labor affects withdrawal behavior has yet to be thoroughly discussed. There needs to be systematic research on how different emotional labor strategies affect work withdrawal, whether directly or through individual perception, and how to respond [7]. Emotional exhaustion, as a reflection of the burnout degree caused by employees’ continuous emotional consumption, will damage employees’ physical and mental health and lead to retreat behavior at work. The impact of emotional labor on job withdrawal may be mediated by emotional exhaustion. It is worth noting that if the emotional resource consumption caused by emotional labor can be effectively supplemented, it will reduce emotional exhaustion caused by resource consumption and avoid work withdrawal behavior [8]. Mindfulness can be regarded as a resource from internal factors. When employees have high mindfulness, they can supplement the psychological resources consumed by emotional labor and relieve the pressure and adverse effects caused by the continuous consumption of their resources [9]. By comprehensively studying the combined effect of individual mindfulness and subjective perception of work burnout experience, the formation mechanism of employees’ work withdrawal behavior can be more effectively analyzed. This paper explores the mediating mechanism of emotional exhaustion between different manifestations of emotional labor and withdrawal behavior and the moderating role of mindfulness between emotional labor, emotional exhaustion, and withdrawal behavior. Our work explores the effective mechanism to intervene in employees’ emotional states and reduce withdrawal behavior. The findings expand the theoretical framework between emotional labor strategies and employees’ withdrawal behavior and provide theoretical support and strategic reference for companies to manage employees’ emotional labor and reduce withdrawal behavior effectively.

## Theoretical background

### Conservation of resources theory

The Conservation of resources (COR) theory proposed by Hobfoll [10] is mainly used to describe the process of resources interacting between individuals and the social environment. The core and fundamental assumptions are that individuals will work hard to preserve, safeguard, and establish valuable resources, including cognitive and capacity resources, and regard their resources’ actual or temporary loss as a threat to themselves. COR theory is widely used in burnout, work stress and anti-productive behavior, work-family conflict, and work performance [11]. Resource exhaustion caused by continuous work pressure and excessive work demands will lead to the emotional exhaustion of individuals. Based on COR, when individual resources are missing, the exhaustion of individual resources can be alleviated if internal or external compensation can be obtained. This provides a theoretical basis for explaining emotional labor, work burnout, and withdrawal behavior.

### Emotional labor and work withdrawal behavior

Emotional labor is defined as “the management of feeling to create a publicly observable facial and bodily display.“ According to the psychological processes of emotional labor, service employees’ two primary emotional labor strategies when interacting with customers are deep acting and surface acting [2]. Surface acting is when the individual hides the genuine emotions in the heart, gives up the due emotional expression and generates the “appropriate” emotions in accordance with the organization’s required display rules, which is a superficial camouflage. Deep acting means that individuals change the existing perception into real feelings by controlling internal thoughts and feelings and genuinely expressing the emotions required by the organization. Surface acting handles emotional expression, while deep acting takes emotions. Deep acting and surface acting affect employees’ physical and mental health, work attitude, and behavior [3].

Withdrawal behavior refers to the various negative behaviors taken by employees to avoid work situations or tasks in an organizational situation [12]. In contrast, career turnover describes leaving a profession or career track [13]. Withdrawal behavior represents a sequence of behaviors that starts from the occasional daydream, gradually expands to being late and absent, and eventually triggers employee turnover. The adverse influence of work withdrawal behavior on employees and institutional effectiveness cannot be overstated. Scholars focus on the content structure and pre-dependent variables, including heavy tasks, hopeless promotion, and interpersonal tension [12, 14], and include individual characteristic variables such as work autonomy, ability, and work preference. Recently, scholars have focused on the impact of employees’ emotional state on withdrawal behavior. Employees who experience more emotional labor may produce work withdrawal behavior caused by emotional labor [14, 15].

In a service-oriented work situation, employees will take emotional labor responses to meet the organization’s requirements for an emotional performance. Although customers’ perception of service quality and satisfaction is improved through emotional labor, employees’ long-term concealment and repression of their emotions will pressure them psychologically and produce a series of work withdrawal behaviors. When employees take surface acting, their internal emotions are far from their external expression. Employees are prone to negative behaviors such as emotional disorders, emotional exhaustion, and work withdrawal behaviors [4]. On the other hand, deep acting is significantly negatively correlated with work withdrawal behavior [14]. Employees who engage in deep acting will significantly reduce their work withdrawal behavior. Deep acting requires employees to adjust their internal emotions and show positive work emotions from the inside out. These consistent internal and external emotions are more likely to resonate with consumers, and employees also feel the sense of achievement brought by work in the process, thus reducing the generation of resignation intention [15]. Therefore, we proposed the following hypotheses:

H1: Surface acting is negatively associated with withdrawal behavior.

H2: Deep acting is positively associated with withdrawal behavior.

### The mediating role of emotional exhaustion

Emotional exhaustion is a state of fatigue that results from using up one’s emotional resources [11]. Emotional exhaustion is the core factor contributing to burnout. This state can be seen as the long-term result of maintaining emotional and interpersonal relationships in the face of inadequate emotional resources. Most studies of burnout use emotional demands as a prerequisite for emotional exhaustion, which is considered to have a unique impact on burnout, which is one of the main consequences of emotional labor [16, 17]. The intervention of emotional labor on work emotional exhaustion is a complex process. Surface acting can lead to resource depletion when employees inhibit the expression of their feelings, generating a discrepancy between their feelings and displayed emotions [2]. In contrast, deep acting is recognized as an adaptive regulation as it is instrumental in obtaining customers’ positive feedback, promoting employees’ efficacy, and giving them a sense of accomplishment [2]. The surface acting of emotional labor positively affects emotional exhaustion and depersonalization, and deep acting negatively influences emotional exhaustion and depersonalization. Deep acting is significantly negatively correlated with withdrawal behavior. Employees who engage in deep acting will significantly reduce their work withdrawal behavior. Deep acting requires employees to adjust their internal emotions and show positive work emotions from the inside out [14]. These consistent internal and external emotions are more likely to resonate with consumers, and employees also feel the sense of achievement brought by work in the process, thus reducing the generation of turnover intention [5, 18]. Emotional exhaustion may mediate the relationship between emotional labor and withdrawal behavior in service settings. Accordingly, the following assumptions are made:

H3: Emotional exhaustion mediates the relationship between surface acting and withdrawal behavior.

H4: Emotional exhaustion mediates the relationship between deep acting and withdrawal behavior.

### The moderating role of mindfulness

Mindfulness is considered a form of intentional, nonjudgmental attention, most defined as involving self-regulation of attention to immediate experience and an open, accepting orientation to that experience [19]. Previous studies primarily focused on the direct effect of mindfulness training on stress reduction. Some studies have shown that employee mindfulness provides optimistic individual-level predictions of performance, organizational and civic behavior, organizational commitment, job satisfaction, and subjective well-being [20]. Mindfulness has also been proven to represent a good predictor of depression, anxiety, stress, and well-being associated with self-compassion, self-efficacy, and gender [21]. Hülsheger et al. found mindful employees are less likely to engage in surface acting, reducing emotional exhaustion and increasing job satisfaction [22]. Similarly, Liang et al. found that mindfulness, through reduced surface acting, was associated with lower levels of supervisor-directed hostility and deviance [23]. In summary, this study shows that engaging in surface acting is resource intensive. It involves demonstrating emotions that are not felt and that focused employees are more inclined to get in touch with their feelings, reducing their reliance on surface acting.

According to COR theory, individuals have an inherent tendency to acquire, conserve, and protect valuable resources in response to resource scarcity. Resource loss caused by emotional labor brings stress and negative attitudes and behaviors such as turnover [10, 11, 24]. Mindfulness, as a supplement to the resource, improves employee work behavior by alleviating emotional consumption. Mindfulness can effectively reduce emotional exhaustion and disorders and reduce work withdrawal behavior caused by burnout [25]. Therefore, we argue that mindfulness may act as a moderator between emotional exhaustion and withdrawal behavior. Mindfulness will have different implications for withdrawal behavior when interacting with emotional exhaustion. Accordingly, the following assumptions are made:

H 5: Mindfulness moderates the effect between emotional exhaustion and withdrawal behavior, such that the relationship is weaker at a higher level of mindfulness.

**Fig. 1:**
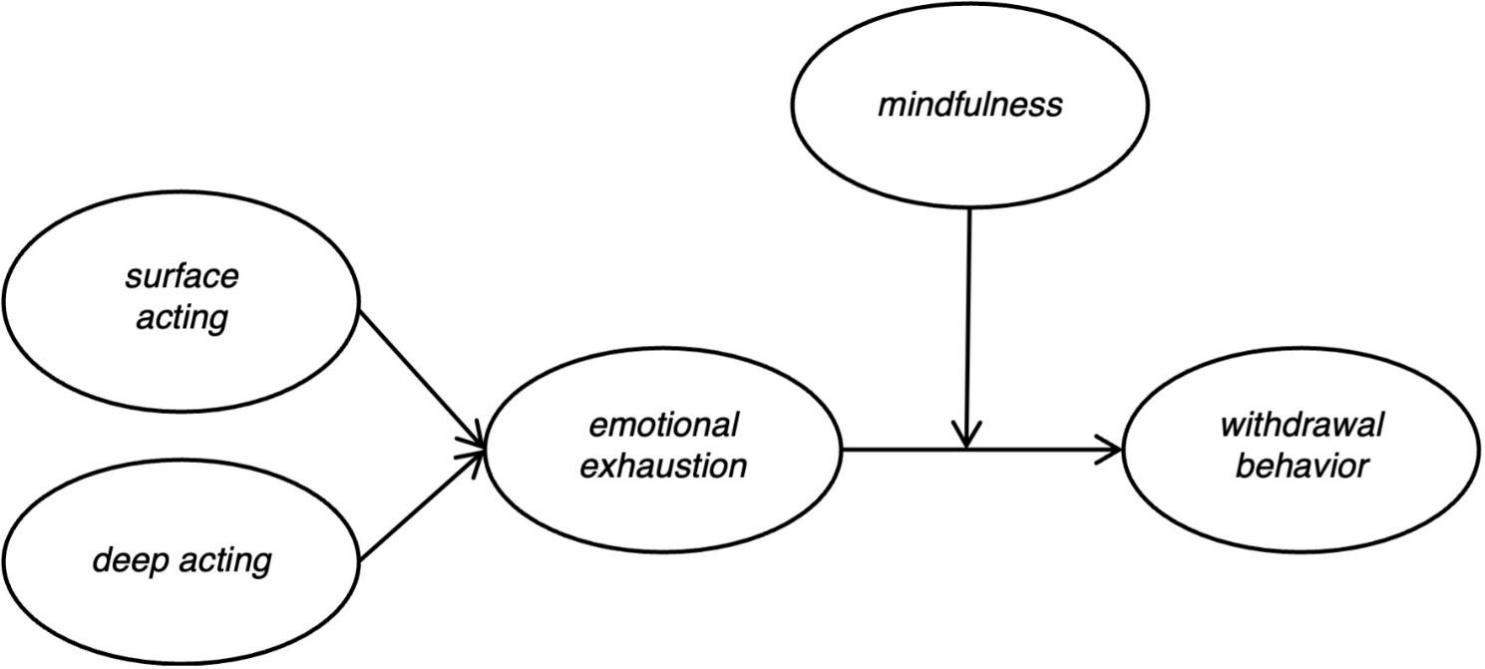
Research model

Based on the previous analysis, the following theoretical model is constructed in this study (see Fig. 1) 

## Materials and methods

### Sample and procedure

This paper combines the random sampling method to emotional labor high incidence of the service industry as the center of the survey. The participants were employees from the hotel and catering industry in China. We contacted the relevant customer service manager and personnel director and distributed the service staff questionnaire with their help. To ensure the quality of the data, we trained the questionnaire-issuing personnel in advance, requiring them to explain the purpose of the survey, and emphasized that the questionnaire information is only used for research purposes and is strictly confidential to ensure that the participants can fill in the questionnaire truthfully and effective. We distributed questionnaires through the online survey. Finally, 286 valid questionnaires were obtained. Of the participants, 104 were men (36.4%), and 182 were women (63.6%). In terms of age, 48 (16.8%) respondents were under 25 years, 80 (28.0%) were within the age bracket of 26–35 years, 141 (49.3%) were within the age bracket of 36–49 years, and 17 (5.9%) were more than 50 years old. In terms of job tenure, 51(17.8%) were less than 5 years, 127 (44.4%) were 5–10 years, and 108(37.8%) participants were over 10 years. Regarding education level, 59.1% held bachelor’s degrees, and 31.1% held postgraduate degrees.

### Measures

All the variables measured in this paper used the mature scale, and the five-point Likert scale was used in the questionnaire design, from significantly disagree to very consent as 1 to 5 points respectively.

*Emotional Labor* Emotional labor is measured using the scale compiled by Glomb & Tews [26]. Surface acting has four questions. Sample items such as “I put on a ‘mask’ to display the emotions I need for the job.” and “I will hide my true feelings to show a specific mood.“ The Cronbach’s α of surface acting was 0.931. Deep acting has five questions: “I will try to overcome bad emotions and sincerely serve the customers with a warm and friendly attitude at work.“ and “I try actually to experience the emotions that I must show customers.” The Cronbach’s α of the deep acting scale was 0.710.

*Emotional Exhaustion* Emotional exhaustion was measured using the scale from the Maslach Burnout Inventory [11]. This scale included 5 items: “I feel emotionally drained from my work.” and “After my work, I regularly feel worn out and weary.“ The Cronbach α was 0.908 in the current study.

*Withdrawal Behavior* The withdrawal behavior of the employees was measured through the eight-item scale developed by Hanisch & Hulin [27]. The specific items were " Left workstation for unnecessary reasons.“ “Put less effort into the job than should have,” etc. The Cronbach’s α coefficient of this scale was 0.936 in the current study.

*Mindfulness* Employee mindfulness was measured with five items developed by Schultz et al. [28]. Sample items include “It seems I am running on automatic, without much awareness of what I am doing” and “The company values my contribution to it.“ The Cronbach’s α coefficient of this scale was 0.942 in the current study.

#### Control variables

We controlled for participants’ gender, age, tenure and education level, all of which have been found to influence employees’ behavior [29].

### Data analysis

Descriptive statistics, confirmatory factor analysis, correlation analysis, and multiple regression analysis were performed using SPSS26.0. Hierarchical regression analysis was conducted to examine the mediation effects of emotional exhaustion according to the recommendations of Baron and Kenny [30]. Finally, SPSS macro program PROCESS was used to test the moderating mediation effect.

## Result

### Descriptive and correlations

All study variables’ means, standard deviations, and correlations are reported in Table 1. A significant positive correlation was found between surface acting and psychological withdrawal behavior (r = 0.543, p < 0.01). Surface acting was also positively correlated with emotional exhaustion (r = 0.322, p < 0.01). Deep acting was found to be negatively related to emotional exhaustion (r = − 0.421, p < 0.01) and withdrawal behavior (r = − 0.321, p < 0.01). Emotional exhaustion positively correlated with withdrawal behavior (r = 0.335, p < 0.01). As anticipated, we found significant correlations between all our central study variables and thus could test the hypothesis.

**Table 1:** Descriptive statistics and correlations among variables (N = 259)

	*M*	*SD*	*1*	*2*	*3*	*4*	*5*	*6*	*7*	*8*
1. Gender	0.36	0.482								
2. Age	2.44	0.839	0.305**							
3. Education	3.21	0.064	− 0.159	0.012						
4. Tenure	3.34	1.559	0.308**	0.665**	− 0.042					
5. Surface acting	3.60	0.681	0.220*	− 0.014	0.056	0.033				
6. Deep acting	2.72	0.935	0.354**	− 0.026	− 0.008	0.036	− 0.219*			
7. Emotional exhaustion	2.81	0.950	− 0.006	− 0.033	0.120	− 0.108	0.322**	− 0.421**		
8. Withdrawal behavior	3.89	0.738	0.089	− 0.053	0.008	0.090	0.543**	− 0.321**	0.335**	
9. Mindfulness	2.45	0.875	− 0.106	− 0.119	− 0.016	− 0.143	0.510**	− 0.287**	0.257**	0.501**

Note. * p < 0.05, ** p < 0.01, gender: 0 = woman, 1 = man

### Measurement model analysis

We conducted a series of confirmatory factor analyses to test the distinctiveness validity of the variables. It was shown that the five-factor measurement model was in good agreement with the actual data (χ^2^/df = 1.93, χ^2^ = 315.33, df = 171, CFI = 0.91, GFI = 0.93, TLI = 0.91, RMSEA = 0.04). It shows that the five variables in the study model have good discriminatory validity. We also compared two four-factor models (respectively: surface acting and deep acting merged into one factor; emotional exhaustion and withdrawal behavior merged into one factor), a three-factor model (surface acting and deep acting merged into one factor, and emotional exhaustion and withdrawal behavior merged into one factor), The five-factor model was found to have the best fit, Better fit over the four-factor model and the three-factor models. As shown in Table 2, the results demonstrate that the theorized five-factor model best fits the data.

**Table 2:** Comparison of measurement models

Models	*χ2*	*df*	*χ2/df*	*CFI*	*GFI*	*TLI*	*RMSEA*
1. Five-factor model	315.33	169	1.86	0.91	0.93	0.91	0.04
2. Four-factor model a	456.85	170	2.68	0.86	0.85	0.87	0.08
3. Four-factor model b	478.75	170	2.81	0.87	0.88	0.89	0.07
4. Three-factor model	551.65	173	3.19	0.80	0.81	0.73	0.09

*Note.* five-factor model: surface acting, deep acting, emotional exhaustion, withdrawal behavior, withdrawal behavior

### Hypotheses testing

#### Main effect

We hypothesized that surface acting positively affects withdrawal behavior(H1). The results are shown in Table 3. Hierarchical regression analyses were performed to test the research hypotheses. In the first step, gender, age, tenure and education level were entered as control variables. After controlling demographic variables, surface acting and deep acting were entered as main effect variables. The results showed surface acting significantly affected withdrawal behavior (model 6: β = 0.542, p < 0.01). Thus, Hypotheses 1a was supported. The relationship between deep acting and withdrawal behavior (H2) was also tested with model 6. The result showed that deep acting significantly negatively affected withdrawal behavior (β = -0.168, p < 0.01). Hypothesis 1b was supported.

#### Mediation effect

To test the intermediary effect of emotional exhaustion, a hierarchical regression analysis test was conducted according to the recommendations of Baron and Kenny [30]. In this method, the establishment of the mediate effect between variables needs to meet three conditions: (1) the influence of the independent variable on the mediator variable reaches a significant level; (2) the influence of the mediator variable on the dependent variable reaches a significant level; and (3) the significance of the independent variable on the dependent variable decreases or disappears due to the addition of the mediator variable. If all three above conditions hold, it can be proved that the influence of the independent variable on the dependent variable is transmitted through the mediator variable.

First, model 2 tested the effect of independent variable emotional labor on emotional exhaustion, and the results showed that surface acting had significant positive effects (β = 0.350, p < 0.01); the deep acting had a significant negative effect on emotional exhaustion (β = -0.395, p < 0.01). Second, the significant effects of surface and deep acting on withdrawal behavior were demonstrated in Model 4, satisfying the first and second conditions of the mediation effect test. Third, put the independent and mediation variables into the regression model 7, examining the changes in the regression coefficients for surface and deep acting. Comparing the regression coefficients of models 6 and 7 shows the effect of surface acting on withdrawal behavior was reduced from the original β = 0.542 (p < 0.01) to β = 0.405 (p < 0.01). This indicates some intermediary utility between surface acting and withdrawal behavior. Thus, H3 is verified. The effect of deep acting on withdrawal behavior was reduced from the original β = -0.168 (p < 0.05) to β = -0.126 (ns). This indicates that emotional exhaustion has a complete intermediary utility between deep acting and withdrawal behavior. The H4 was validated.

**Table 3:** Results of hierarchical regression analysis

	*Emotional exhaustion*				*Withdrawal behavior*				
	*model 1*	*model 2*	*model 3*	*model 4*	*model 5*	*model 6*	*model 7*	*model 8*	*model 9*
Constant	2.346	2.467	2.282	2.146	3.857	2.610	2.347	3.582	2.110
Gender	0.084	− 0.097	− 0.137	-0.95	0.149	− 0.072	− 0.061	0.125	0.094
Age	0.063	0.084	0.094	0.102	− 0.202	− 0.154	− 0.163	− 0.227*	− 0.224*
Education	0.186	0.128	0.127	0.127	0.042	− 0.027	− 0.040	− 0.022	− 0.016
Tenure	− 0.094	− 0.124	− 0.124	− 0.130	0.101	0.082	0.095	0.122	0.116
SA		0.350**	0.311*	0.378*		0.542**	0.405**		
DA		− 0.395**	-382**	− 0.391**		− 0.168*	− 0.126		
MI			0.077**	0.069				− 0.095	− 0.072
EE							0.206*	0.258	0.570**
SA *MI				− 0.272					
DA*MI				− 0.060					
EE*MI									− 0.180*
R^2^	0.029	0.274	0.277	0.303	0.039	0.360	0.374	0.172	0.204
△R^2^	0.029	0.246	0.003	0.026	0.039	0.321	0.014	0.009	0.032
△F	0.773	17.426**	0.442	1.871	1.077	25.806**	2.218	1.088	4.408*

Note. *p < 0.05, **p < 0.01, SA = surface acting, DA = deep acting, EE = emotional exhaustion, MI = mindfulness, gender: male = 1, female = 0

#### Moderating effect

**Fig. 2:**
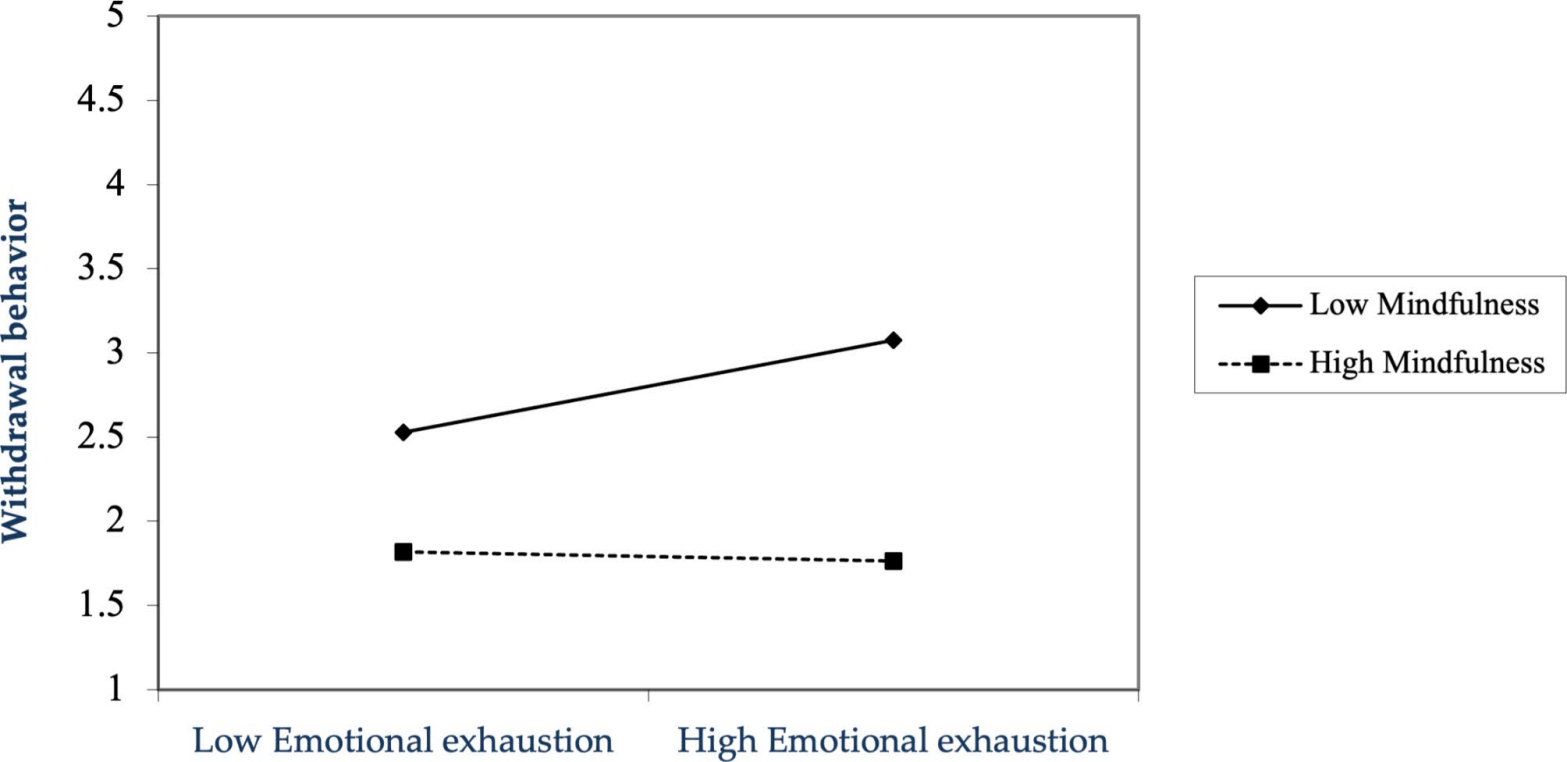
Moderating effect of mindfulness on the relationship between emotional exhaustion and withdrawal behavior

In addition, we suggested that mindfulness would regulate the relationship between emotional exhaustion and withdrawal behavior (H5). We used hierarchical regression to examine the moderating effect. Withdrawal behavior was first set as the dependent variable, and the control variable, emotional exhaustion, and the moderator variable, mindfulness, were introduced. Finally, a centralized interaction term for emotional exhaustion and mindfulness was added in model 9. The results show that the model interpretation ability improved significantly (△R^2^ = 0.032, p < 0.05). The interaction term of emotional exhaustion and mindfulness significantly impacted withdrawal behavior (β = -0.180, p < 0.05), indicating a significant adverse moderating effect of mindfulness on the relationship between emotional exhaustion and withdrawal behavior. H5 was validated. That implies that employees with greater degrees of mindfulness tend to exhibit lower withdrawal behavior. Employees with higher levels of mindfulness are much more likely to reduce their work withdrawal behavior by eliminating emotional exhaustion when faced with emotional labor. The results of the moderating effect of employees’ mindfulness on the relationship between emotional exhaustion and withdrawal behavior are depicted in Fig. 2.

#### Moderated mediation

In addition, Hypothesis 4 proposes that mindfulness will change the mediating effect of emotional exhaustion between emotional labor strategies and withdrawal behavior. To test this hypothesis, according to the suggestions of Edwards and Lambert [31], we use the bootstrapping method to analyze the changes in the mediating effect of emotional exhaustion at work between emotional labor and withdrawal behavior at different mindfulness levels, using surface acting and deep acting as independent variables. The analysis results with the surface acting as a dependent variable are shown in Table 4. When mindfulness is low, the positive impact of the first stage (effect of surface acting on emotional exhaustion) is significant (β = 0.34, p < 0.01). The positive effect of the first stage is also significant when mindfulness is high (β = 0.29, p < 0.01), but there is no significant difference between them (β = 0.05, ns). The positive effect of the second stage (effect of emotional exhaustion on withdrawal behavior) is significant when mindfulness is low (β = 0.46, p < 0.01), and the positive effect of the second stage is also significant when mindfulness is high (β = 0.27, p < 0.01), and the difference between high and low mindfulness in the second stage is also significant (β = 0.19, p < 0.01). The regulatory role of mindfulness is mainly reflected in the path from emotional exhaustion to withdrawal behavior. In addition, there is no significant difference between low mindfulness and high mindfulness direct effects, but a significant difference in indirect effects (β = 0.14, p < 0.05). Mindfulness has a significant regulatory effect on the mediating effect of emotional exhaustion on the relationship between surface acting and withdrawal behavior, and H4a has been verified.

**Table 4:** Moderated mediation test-taking surface acting as the mediator

Moderator variable	SA(X) → EE(M) → WB(Y)				
	Step1	Step2	Direct	Indirect	Total
Low-Mindfulness	0.34**	0.46**	0.15*	0.26**	0.41**
High-Mindfulness	0.29**	0.27**	0.17*	0.12*	0.29**
Difference	0.05	0.19**	0.02	0.14*	0.12**

Note. *p < 0.05, **p < 0.01, SA = surface acting, EE = emotional exhaustion, WB = withdrawal behavior, Step1 = effect of sur-face acting on emotional exhaustion, Step2 = effect of emotional exhaustion on withdrawal behavior

The analysis results of deep acting as a dependent variable are shown in Table 5. When mindfulness is low, the negative impact of the first stage is significant (β = − 0.32, p < 0.01), and the negative effect of the first stage is also significant when mindfulness is high (β = − 0.27, p < 0.01), but there is no significant difference between them (β = 0.05, ns). When mindfulness is low, the positive effect of the second stage is significant (β = 0.43, p < 0.01), the positive effect of the second stage is also significant when mindfulness is high (β = 0.23, p < 0.01), and the difference between high and low mindfulness in the second stage is also significant (β = 0.20, p < 0.01). The regulatory role of mindfulness is mainly reflected in the path from emotional exhaustion to withdrawal behavior. In addition, there is no significant difference in direct effects between low mindfulness and high mindfulness, but a significant difference in indirect effects (β = 0.11, p < 0.05). Mindfulness has a significant regulatory effect on the mediating effect of emotional exhaustion in the relationship between deep acting and withdrawal behavior. H4b has been verified.

**Table 5:** Moderated mediation test-taking deep acting as the mediator

Moderator variable		DA(X)	→ (M)	→ WB(Y)	
	Step1	Step2	Direct	Indirect	Total
Low-Mindfulness	-0.32**	0.43**	0.09	0.25**	0.34*
High-Mindfulness	-0.27**	0.23**	0.08	0.14*	0.22**
Difference	0.05	0.20**	0.01	0.11*	0.12**

Note. *p < 0.05, **p < 0.01, DA = deep acting, EE = emotional exhaustion, WB = withdrawal behavior

## Discussion

Based on the COR theory, this study examined the relationship between emotional labor, emotional exhaustion, mindfulness, and withdrawal behavior. The COR theoretical framework suggests people are inspired to preserve and avoid losing resources. From this perspective, we sought to investigate how mindfulness can help employees retain their resources and avoid resource loss when faced with high-intensity work demands. According to our findings, mindfulness may be a protective factor, independent of the intensity of an individual’s emotional labor stress. The improvement of mindfulness level can effectively relieve emotional exhaustion and withdrawal behavior.

The study found that employees’ emotional labor not only affects customers’ perception of service quality but also affects employees’ work behavior. The surface acting of emotional labor positively affects employees’ withdrawal behavior. In contrast, deep acting behavior negatively affects withdrawal behavior, which is consistent with the research results of Scott & Barnes [14]. When employees take the surface acting, their internal feelings are far from their external expression, which can have negative effects such as emotional disorders and withdrawal behaviors. Deep acting requires employees to adjust their internal emotions and show positive work emotions from the outside. Employees also feel the sense of achievement brought by work in the process, thus reducing the generation of withdrawal behavior [3]. That is to say, the tendency of employees to work retreat behavior increases significantly during surface acting, while the tendency of work retreat behavior is reduced when performing deep acting. This shows that the role of emotional labor on work withdrawal behavior is a complex project. Surface acting will cause a particular emotional consumption. If there is no relief and supplement, the more emotional resource is depleted, the more likely it is to cause withdrawal behavior. At the same time, deep acting can relieve pressure and accumulate positive emotions, thus reducing the generation of work withdrawal behavior.

Secondly, we introduced the concept of emotional exhaustion to the research model, revealing the mediating mechanism of emotional labor to work withdrawal behavior. It was found that the impact of different emotional labor strategies on withdrawal behavior can be mediated by emotional exhaustion. The results of the intermediary transmission effect of emotional exhaustion between the surface play and the work withdrawal behavior are consistent with the emotional exhaustion results verified by Scott & Barnes [14].

In addition, in revealing the impact mechanism of emotional labor on withdrawal behavior, the moderating effect of mindfulness is verified to make the impact mechanism between emotional labor and work withdrawal behavior further accurate and controllable. The study found that mindfulness is closely related to emotional exhaustion and withdrawal behavior and that mindfulness negatively regulates the relationship between emotional exhaustion and withdrawal behavior. Employees with higher mindfulness find it easier to eliminate emotional exhaustion in the face of emotional labor and thus reduce their work withdrawal behavior.

### Theoretical contributions

Our study makes significant contributions to the emotional labor and withdrawal behavior literature. First, based on COR theory, we have accounted for the different actions of various emotional labor strategies as antecedents of employee withdrawal behavior. Moreover, we verify the mediation of emotional exhaustion between emotional labor and withdrawal behavior. Although emotional labor has been linked to employee performance and this relationship has been theorized and discussed (), little research has demonstrated how emotional exhaustion can mediate the effect of emotional labor on withdrawal behavior. The primary intermediary role of emotional exhaustion in the influence of surface and deep acting on employee withdrawal behavior has led to a more precise mechanism of action between emotional labor and withdrawal behavior. These findings extend the current understanding of the effects of emotional labor, and the results have significant consequences for the development of emotional labor theory, especially in relation to employee withdrawal behavior.

Second, we extended the understanding of the boundary of employee mindfulness in emotional labor situations by highlighting its moderation role in the positive relationship between emotional exhaustion and withdrawal behavior. This study verifies the moderation effect of mindfulness, which supplements the psychological resources consumed by emotional labor and alleviates the stress and adverse effects caused by the continuous consumption of own resources [9]. The results echo earlier literature, showing that mindfulness represents a good predictor of depression, anxiety, stress, and well-being [21] [22]. According to the theory of COR, it is a beneficial expansion of the study of emotional labor and withdrawal behavior, which provides a basis for future research from the perspective of resource supplements.

### Practical implications

This study certainly has practical implications as well. Regarding management practice, the research results provide new ideas and perspectives to solve the withdrawal behavior of emotional labor employees. Employees often review what work means to them and how much space it should occupy. Our research results show that emotional labor strategies significantly impact withdrawal behavior. Therefore, enterprises should not only consider environmental factors such as work difficulty and salary but also analyze the emotional loss and pay attention to the emotional status of employees. The adverse impact of emotional labor mainly comes from surface acting, and the positive effect comes from deep acting. Managers should adopt various strategies to guide and train employees to use more deep acting, such as creating an excellent working atmosphere and establishing harmonious communication mechanism to promote good interaction between employees and customers; providing psychological counseling to employees with surface acting tendencies, give their emotional adjustment time, and then participate in the work after adjustment.

Moreover, emotional exhaustion has become an essential negative factor affecting employees’ physical and mental health and work efficiency and alleviating the negative impact of emotional exhaustion has become managers’ focus. Managers should enhance the emotional interaction with employees as much as possible, master employees’ practical state, and embody the “people-oriented” management philosophy. In addition, the study found that mindfulness significantly inhibits work withdrawal behavior caused by emotional exhaustion, which shows that employees can try to relieve their burnout through mindfulness and reduce the risk of silent resignation from the perspective of cultivating and improving their mindfulness level. The enterprise can enhance the self-efficacy and autonomy of employees, emphasize the significance and value, and adopt corresponding incentive factors to enhance the mindfulness level of employees, thus effectively reducing the possible withdrawal behavior caused by emotional exhaustion.

### Limitations and future directions

The study has the following limitations, which need to be further explored. First, the survey of this paper mainly focuses on hotel, catering, and other emotional labor higher industries. Research conclusion applicability has certain limitations for other regions and industries. Future studies may employ a longitudinal method of gathering statistics from different fields. In addition, this paper focuses on adjusting the negative impact of emotional labor on the employee level, but the different emotional labor strategies have completely different results. We found that deep acting can bring many positive effects, so the subsequent research on how to guide surface acting to deep acting is a future research priority.

Secondly, this paper has examined the intermediary impact of emotional exhaustion. The influence of emotional labor on withdrawal behavior may also be mediated through the emotional state, pressure, and other variables. Future research can further introduce other individual-level intermediary variables to study the mediating mechanism of emotional labor and employee withdrawal behavior.

In addition, the study of withdrawal behavior has reference significance for understanding the phenomenon of quiet quitting, but quiet quitting is not identical to work withdrawal. In the future, we can compare quiet quitting and withdrawal behavior and explore new features of quiet quitting.

## Conclusions

This paper combines the resource conservation theory to reveal the emotional labor mechanism on work withdrawal behavior. Studies have shown that different levels of emotional labor influence employee withdrawal behavior. Employees’ tendency to work retreat behavior obviously increases during surface acting but can reduce the tendency of work withdrawal behavior when deep acting. In addition, emotional exhaustion mediates the relation between emotional labor and withdrawal behavior, and mindfulness negatively regulates the withdrawal behavior caused by emotional exhaustion. If employees can self-regulate mindfulness and enhance autonomy, responsibility, and self-determination, supplement psychological resources and reduce the negative effects of emotional exhaustion on withdrawal behavior. The research conclusions further enrich the theoretical understanding of emotional labor and provide inspiration for the employee management of service-oriented enterprises.

## Data Availability

The raw data supporting the conclusions of this article will be made available by the authors, without undue reservation.
